# Ca_16_Mab-56, a Novel Anti-Cadherin-16/Ksp-Cadherin Monoclonal Antibody for Multiple Applications

**DOI:** 10.3390/antib15050083

**Published:** 2026-09-07

**Authors:** Yukari Ogura, Hiroyuki Suzuki, Mika K. Kaneko, Yukinari Kato

**Affiliations:** Department of Antibody Drug Development, Tohoku University Graduate School of Medicine, 2-1 Seiryo-machi, Aoba-ku, Sendai 980-8575, Miyagi, Japan; ogura.yukari.t4@dc.tohoku.ac.jp (Y.O.); mika.kaneko.d4@tohoku.ac.jp (M.K.K.)

**Keywords:** monoclonal antibody, Cell-Based Immunization and Screening, cadherin-16, Ksp-cadherin, flow cytometry, immunohistochemistry

## Abstract

Background/Objectives: Cadherin-16 (CDH16, Ksp-cadherin) possesses unique seven extracellular cadherin repeats, and its expression is restricted to normal kidney epithelium. CDH16 is downregulated in renal cell carcinoma (RCC) and is associated with poor prognosis. Therefore, developing mAbs that specifically recognize cell-surface CDH16 is essential for tumor diagnosis and for isolating CDH16-positive renal epithelial cells. Methods: Anti-human CDH16 mAbs (designated as Ca_16_Mabs) were developed by immunizing mice with CDH16-overexpressed tumor cells, followed by a high-throughput flow cytometry-based screening. Results: Among the 58 established Ca_16_Mabs, a clone, Ca_16_Mab-56 (IgG_1_, κ), specifically recognized CDH16-overexpressed Chinese hamster ovary-K1 (CHO/CDH16) cells with no detectable cross-reactivity to 21 other CDHs in flow cytometry. Ca_16_Mab-56 also detected endogenous CDH16 in human RCC cell lines (OS-RC-2 and KMRC-20) and normal kidney epithelial cell lines. The dissociation constant (*K*_D_) values of Ca_16_Mab-56 for CHO/CDH16 and OS-RC-2 were determined as 6.5 × 10^−9^ M and 1.2 × 10^−9^ M, respectively. Furthermore, Ca_16_Mab-56 detected endogenous CDH16 by Western blotting and showed potent staining in normal kidney tubular epithelium and clear membranous staining in renal cell carcinoma in immunohistochemistry. Conclusions: Ca_16_Mab-56 is a versatile tool for detecting CDH16 and has potential for tumor diagnosis.

## 1. Introduction

Cadherins (CDHs) are essential mediators of cell–cell adhesion and are required for the maintenance of tissue architecture [1]. Classical cadherins are characterized by five extracellular cadherin (EC) repeats, a single-pass transmembrane domain, and a cytoplasmic tail containing highly conserved binding sites for armadillo-family proteins, including β-catenin and p120-catenin [1]. The EC repeats, first characterized in the extracellular domain of the classical type I cadherin CDH1/E-cadherin [2], coordinate Ca^2+^ ions and mediate homophilic binding, a property critical for the sorting of cells into distinct compartments during organogenesis [3]. Within epithelial tissues, CDH1 assembles adherens junctions through homophilic interactions, thereby establishing the defining architectural and apical adhesive properties of epithelial sheets [4]. A defining functional feature of classical cadherins is their capacity to engage cytoplasmic armadillo proteins: p120-catenin directly binds to the membrane-proximal region of the cadherin cytoplasmic domain, while β-catenin functions as a scaffold that recruits α-catenin [5]. The resulting cadherin–catenin complex interacts, directly or indirectly, with an array of signaling molecules, scaffolding proteins, and cytoskeletal regulators, most notably filamentous actin [5].

The seven-domain cadherin (7D cadherin) subfamily comprises two members, CDH16 (kidney-specific cadherin; Ksp-cadherin) and its paralog CDH17 (liver-intestine cadherin; LI-cadherin) [6]. These CDHs are distinguished by a tandem duplication of the first two EC repeats, resulting in seven EC repeats instead of the five found in classical CDHs [7]. In addition, both proteins possess truncated cytoplasmic domains that lack the armadillo protein-binding motifs characteristic of classical CDHs [1].

Although CDH16 and CDH17 share similar structural features, they exhibit distinct expression patterns and biological functions. CDH16 is expressed on the basolateral membrane of epithelial cells lining the nephron and the collecting system of the mammalian kidney [8,9]. CDH16-deficient mice were viable and fertile, with no apparent renal structural abnormalities or defects in epithelial polarity. Renal function remained largely normal, although the mice exhibited a transient impairment in maximal urinary concentrating ability, likely due to altered aquaporin-2 expression in the inner medullary collecting duct [10]. Since CDH16 expression is restricted to the kidney, the promoter region was extensively analyzed [11,12]. The CDH16 promoter-Cre mice were developed [13,14] and have been utilized in gene modification in the kidney [15,16,17].

CDH16 is frequently downregulated in malignancies. In normal tissues, CDH16 is expressed in renal epithelial cells, but its expression is reduced in renal cell carcinomas (RCCs) [8,18,19]. The downregulation of CDH16 is linked to poor prognosis in clear cell RCC [20]. CDH16 is also expressed in the thyroid gland, where its downregulation occurs early during thyroid carcinogenesis, preceding the loss of CDH1 in some cases [21]. Furthermore, transforming growth factor-β induces epithelial–mesenchymal transition accompanied by decreased CDH16 expression in thyroid cells [21]. Although CDH16 appears to play an important role in kidney and thyroid development, the molecular mechanisms underlying its tumor-suppressive function remain poorly understood.

MAbs that detect CDH16 have been developed for Western blotting or immunohistochemistry (IHC). These anti-CDH16 mAbs recognize the C-terminal cytoplasmic domain of CDH16 (e.g., clone 4H6/F9 [18,20,22]), or the detailed immunogens remain unknown [20,23]. However, information on cross-reactivity with other CDHs is unavailable. Moreover, anti-CDH16 mAbs suitable for both flow cytometry and IHC are limited. We have developed the Cell-Based Immunization and Screening (CBIS) method. The method includes the immunization of antigen-overexpressed cells and high-throughput flow cytometry-based screening. MAbs established by the CBIS method recognize conformational epitopes and are applicable in flow cytometry. Importantly, some of them are also suitable for Western blotting and IHC. Using the CBIS method, we have developed anti-CDH1 [24], anti-CDH13 [25], anti-CDH15/M-cadherin [26], and anti-CDH17 [27] mAbs for flow cytometry, Western blotting, and IHC. In this study, we developed anti-CDH16 mAbs by the CBIS method and selected a specific and highly versatile anti-CDH16 mAb.

## 2. Materials and Methods

### 2.1. Cell Lines

Human glioblastoma (GBM) LN229, Chinese hamster ovary (CHO)-K1, immortalized human normal kidney epithelial RPTEC/TERT1, and mouse myeloma P3X63Ag8U.1 (P3U1) cell lines were obtained from the American Type Culture Collection (ATCC, Manassas, VA, USA). The human RCC cell line OS-RC-2 was obtained from the RIKEN BioResource Research Center (Ibaraki, Japan). Another RCC cell line, KMRC-20, was obtained from the Japanese Collection of Research Bioresources (JCRB) Cell Bank (Osaka, Japan). CHO-K1, P3U1, and CDH-overexpressed CHO-K1 (e.g., CHO/CDH16), OS-RC-2, KMRC-20, LN229, and CDH16-overexpressed LN229 were cultured as described previously [24]. RPTEC/TERT1 was cultured as described previously [28].

### 2.2. Establishment of Cadherin-Overexpressed Stable Transfectants

Full-length CDH16 cDNA (NM_004062) was obtained from RIKEN BioResource Research Center. The signal sequence-deleted CDH16 cDNAs with an N-terminal MAP16 tag (PGTGDGMVPPGIEDKI) was subcloned into the pCAG-Ble vector (FUJIFILM Wako Pure Chemical Corporation, Osaka, Japan). The signal sequence-deleted CDH16 cDNAs with an N-terminal PA16-tag (GLEGGVAMPGAEDDVV) were subcloned into the pCAG-Ble vector. These vectors were transfected into LN229 or CHO-K1 cells using the Neon transfection system (Thermo Fisher Scientific, Inc., Waltham, MA, USA). Stable transfectants were sorted using an anti-PA16-tag mAb (clone NZ-1) or an anti-MAP16 tag mAb (clone PMab-1). Finally, MAP16-CDH16-overexpressed LN229 (LN229/CDH16) and PA16-CDH16-overexpressed CHO-K1 (CHO/CDH16) were established.

Another 7D CDH-overexpressed CHO-K1: CHO/CDH17 (CHO/CDH17), a truncated CDH-overexpressed CHO-K1: CHO/CDH13 (CHO/PA16-CDH13), and an atypical CDH -overexpressed CHO-K1: CHO/CDH26 (CHO/PA16-CDH26) were previously established [29]. Type I CDH-overexpressed CHO-K1: CHO/CDH1, CHO/CDH2 (CHO/PA16-CDH2), CHO/CDH3, CHO/CDH4 (CHO/PA16-CDH4), and CHO/CDH15 (CHO/PA16-CDH15) were previously established [24]. Type II CDH-overexpressed CHO-K1: CHO/CDH5 (CHO/PA16-CDH5), CHO/CDH6, CHO/CDH7 (CHO/PA16-CDH7), CHO/CDH8 (CHO/PA16-CDH8), CHO/CDH9 (CHO/PA16-CDH9), CHO/CDH10 (CHO/PA16-CDH10), CHO/CDH11 (CHO/PA16-CDH11), CHO/CDH12 (CHO/PA16-CDH12), CHO/CDH18 (CHO/PA16-CDH18), CHO/CDH19 (CHO/PA16-CDH19), CHO/CDH20 (CHO/PA16-CDH20), CHO/CDH22 (CHO/PA16-CDH22), and CHO/CDH24 (CHO/PA16-CDH24) were established previously [29].

Each cadherin expression was confirmed using an anti-CDH1 mAb (clone Ca_1_Mab-3), an anti-CDH3 mAb (clone MM0508-9V11, Abcam, Cambridge, UK), an anti-CDH6 mAb (clone 427909, R&D Systems Inc., Minneapolis, MN, USA), an anti-CDH8 mAb (clone Ca_8_Mab-4), an anti-CDH16 mAb (Code: BT-MCA3873, BT LAB, Zhejiang, China), an anti-CDH17 mAb (clone Ca_17_Mab-5 [27]), and an anti-PA16-tag mAb (clone NZ-33) to detect other cadherins with a PA16-tag.

### 2.3. Hybridoma Production

All animal procedures were conducted in accordance with applicable guidelines and regulations, with measures to minimize pain, distress, and suffering. The experimental protocol was reviewed and approved by the Animal Care and Use Committee of Tohoku University (Permit No. 2019NiA-001). The mice were maintained under specific pathogen-free conditions and on an 11 h light/13 h dark cycle throughout the experiment. Food and water were provided ad libitum. The mice were monitored daily for general health and well-being throughout the experiment. A reduction in body weight exceeding 25% of the initial body weight was predetermined as a humane endpoint. The mice were euthanized by cervical dislocation, and death was confirmed by the cessation of both respiration and cardiac activity. Two female BALB/cAJcl mice (6 weeks old; CLEA Japan, Tokyo, Japan) were used for immunization. Each mouse received an intraperitoneal injection of 1 × 10^8^ LN229/CDH16. The primary immunization was administered with 2% Alhydrogel adjuvant (InvivoGen, San Diego, CA, USA), whereas the subsequent three weekly immunizations were performed without adjuvant. A final booster injection of the same number of LN229/CDH16 cells was given intraperitoneally 2 days before spleen harvest. Hybridoma generation was carried out according to a previously established protocol [23]. To identify CDH16-reactive clones, hybridoma supernatants were initially screened by flow cytometry using CHO/CDH16 together with parental CHO-K1 as a negative control. One selected mAb, Ca_16_Mab-56, was purified from hybridoma culture supernatant maintained in serum-free Hybridoma-SFM medium (Thermo Fisher Scientific, Inc.) using Ab-Catcher Extra affinity resin (ProteNova, Higashikagawa, Japan).

### 2.4. Flow Cytometry

Cells were harvested with 1 mM EDTA and washed with blocking buffer [0.1% bovine serum albumin in phosphate-buffered saline (PBS)]. The cells were incubated with primary mAbs for 30 min at 4 °C. The cells were then stained with Alexa Fluor 488-conjugated anti-mouse IgG (1:2000; Cell Signaling Technology, Inc., Danvers, MA, USA). Flow cytometric data were acquired on an SA3800 Cell Analyzer (Sony Corp., Tokyo, Japan). Cells were gated on forward scatter (FSC) and side scatter (SSC), and fluorescence intensity was analyzed using FlowJo software (Ver.10.8.1, BD Biosciences, Franklin Lakes, NJ, USA).

### 2.5. Calculation of the Binding Affinity by Flow Cytometry

To evaluate the binding affinity of Ca_16_Mab-56, cells were incubated with serially diluted mAbs, followed by staining with Alexa Fluor 488-conjugated anti-mouse IgG (1:200 dilution). Fluorescence signals were acquired by flow cytometry, and the geometric mean fluorescence intensity (GeoMean) was analyzed using FlowJo software. The dissociation constant (*K*_D_) was estimated by fitting the binding curves (antibody concentration versus GeoMean) to a one-site binding model implemented in GraphPad Prism 6 (GraphPad Software, Inc., La Jolla, CA, USA).

### 2.6. Western Blotting

Western blotting was performed as described previously [25]. Ca_16_Mab-56 (1 μg/mL), BT-MCA3873 (1 μg/mL), and an anti-isocitrate dehydrogenase 1 (IDH1) mAb (clone RcMab-1-mG_1_, 2 μg/mL) were used as primary mAbs. Horseradish peroxidase-conjugated anti-mouse IgG (1:1000; Agilent Technologies Inc., Santa Clara, CA, USA) was used as secondary mAb. Chemiluminescence signals were developed using Pierce™ ECL Plus (Thermo Fisher Scientific, Inc.) or ImmunoStar LD (Wako Pure Chemical Corporation). The signals were imaged with ChemiDoc Touch MP (Bio-Rad Laboratories, Inc., Berkeley, CA, USA).

### 2.7. Immunohistochemistry Using Cell Blocks and Tissue Microarrays

IHC was performed using the VENTANA BenchMark ULTRA PLUS (Roche Diagnostics, Indianapolis, IN, USA). CHO/CDH16, CHO-K1, and OS-RC-2 were fixed with formaldehyde, and cell blocks were prepared using iPGell (Genostaff Co., Ltd., Tokyo, Japan). Formalin-fixed, paraffin-embedded (FFPE) cell sections were stained with Ca_16_Mab-56 (1 or 5 μg/mL), BT-MCA3873 (0.2 μg/mL), or CvMab-62 (5 μg/mL, IgG_1_ isotype control). A multiple kidney tumor microarray (KD1501a, US Biomax Inc., Rockville, MD, USA) and a human normal kidney tissue array (NC07-01-001, Cybrdi, Rockville, MD, USA) were stained with Ca_16_Mab-56 (5 μg/mL). Staining was performed using the ultraView Universal DAB Detection Kit (Roche Diagnostics).

## 3. Results

### 3.1. Development of Anti-CDH16 mAbs by the CBIS Method

**Figure 1:**
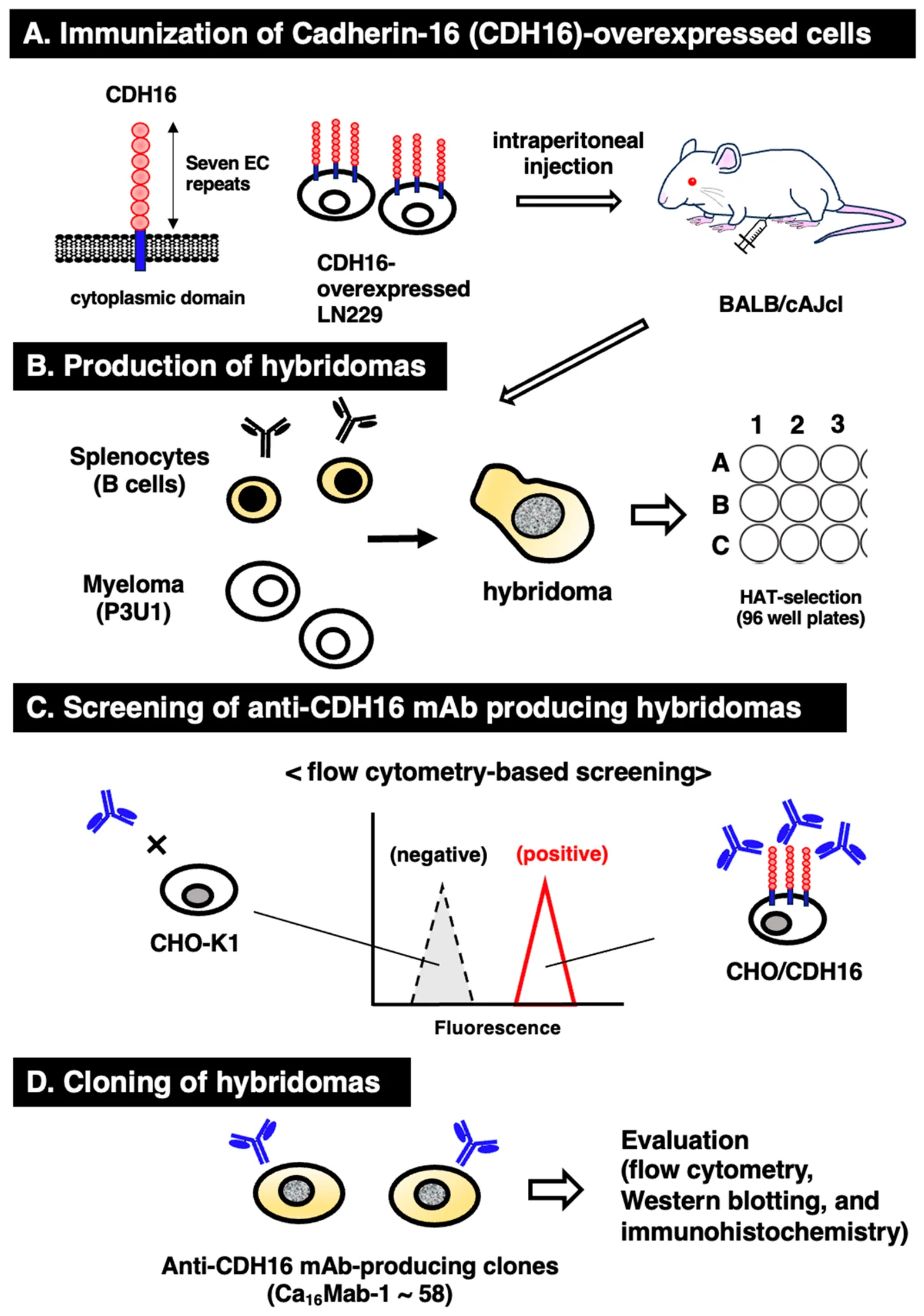
Development of anti-CDH16 mAbs. (**A**) LN229/CDH16, CDH16-overexpressed LN229, was used as an immunogen and injected into two BALB/cAJcl mice. (**B**) The splenocytes were fused with P3U1 after five immunizations. (**C**) Flow cytometry-based screening identified hybridoma supernatants positive for CHO/CDH16 and negative for CHO-K1. (**D**) Using limiting dilution, anti-CDH16 mAb-producing hybridoma clones (Ca_16_Mabs) were established and evaluated for applications.

As described in Section 2.2 and Figure 1A, LN229/CDH16 was established as an immunogen. Two BALB/cAJcl mice were immunized with LN229/CDH16 (1 × 10^8^ cells) intraperitoneally (Figure 1A). After five immunizations, the splenocytes were fused with myeloma P3U1 cells, which were plated in 96-well plates (Figure 1B). At 6 days after the fusion, hybridoma supernatants were screened to identify those positive for CHO/CDH16 and negative for CHO-K1 (Figure 1C). The screening identified 218 out of 948 wells (23%) that showed strong reactivity to CHO/CDH16 compared with CHO-K1. Limiting dilution was then conducted, and 58 anti-CDH16 mAb-producing clones were established. Using the supernatants of established clones, we further evaluated their utility in flow cytometry, Western blotting, and IHC (Figure 1D). Finally, clone Ca_16_Mab-56 (IgG_1_, κ) was selected because it can be used in all applications (https://www.med-tohoku-antibody.com/topics/antibody_bank.htm, accessed on 21 July 2026).

### 3.2. Flow Cytometric Analysis of Ca_16_Mab-56 Against CHO-K1 and CHO/CDH16

**Figure 2:**
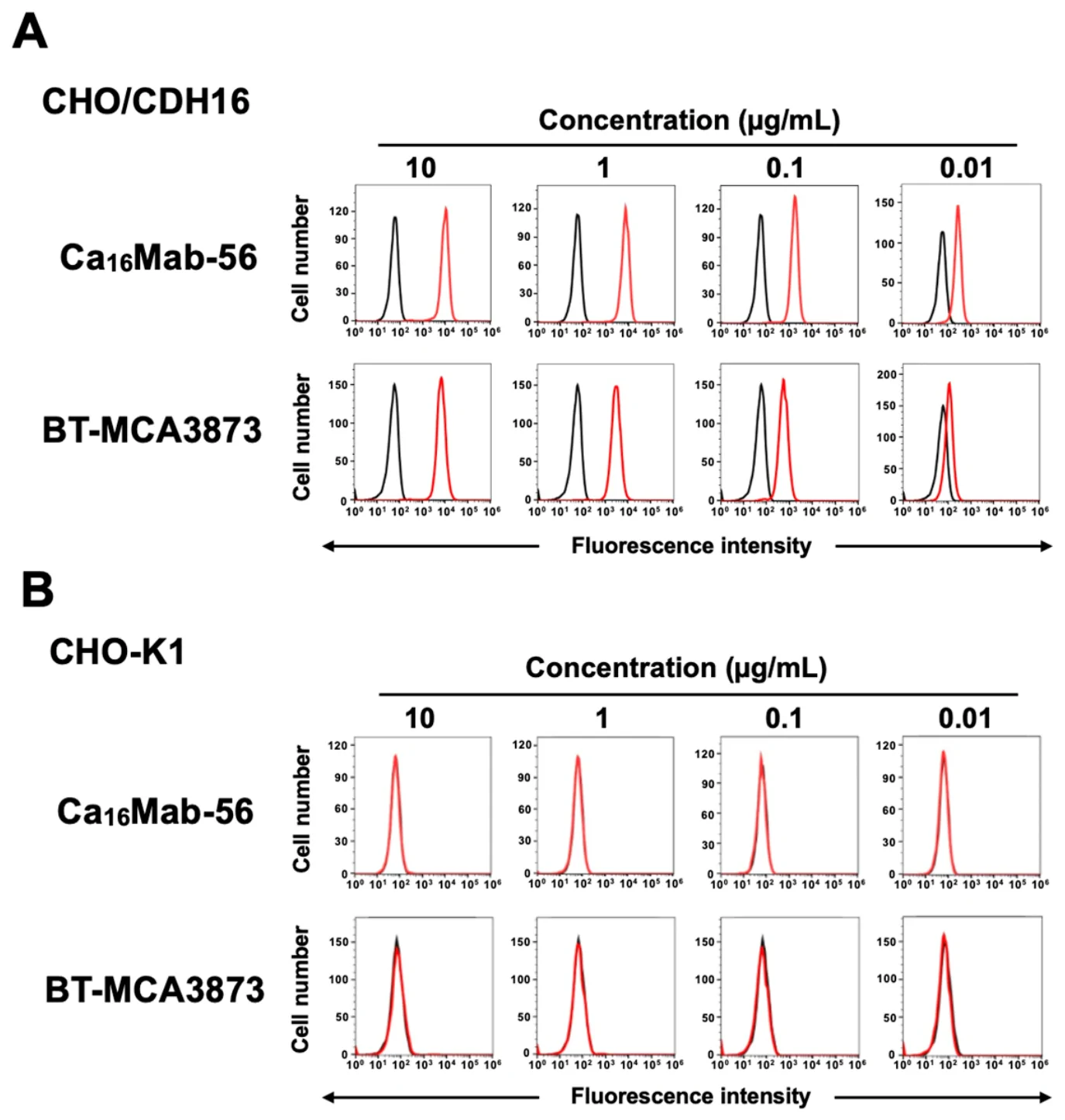
Flow cytometric analysis using Ca_16_Mab-56 and BT-MCA3873. CHO/CDH16 (**A**) and CHO-K1 (**B**) were treated with Ca_16_Mab-56 or BT-MCA3873 at 10, 1, 0.1, and 0.01 μg/mL (red line) or blocking buffer (black line, negative control). These cells were further treated with Alexa Fluor 488-conjugated anti-mouse IgG. Fluorescence data were collected using the SA3800 Cell Analyzer. The experiments were conducted twice.

Using purified Ca_16_Mab-56, reactivity with CHO/CDH16 or parental CHO-K1 was assessed by flow cytometry. Ca_16_Mab-56 dose-dependently recognized CHO/CDH16 from 10 to 0.01 μg/mL (Figure 2A). In contrast, Ca_16_Mab-56 did not react with CHO-K1 even at 10 μg/mL (Figure 2B). A commercially available anti-CDH16 mAb (BT-MCA3873) also reacted with CHO/CDH16 in a dose-dependent manner (Figure 2A) but did not recognize CHO-K1 (Figure 2B).

### 3.3. The Specificity of Ca_16_Mab-56 and BT-MCA3873 Using CDHs-Overexpressed CHO-K1

**Figure 3:**
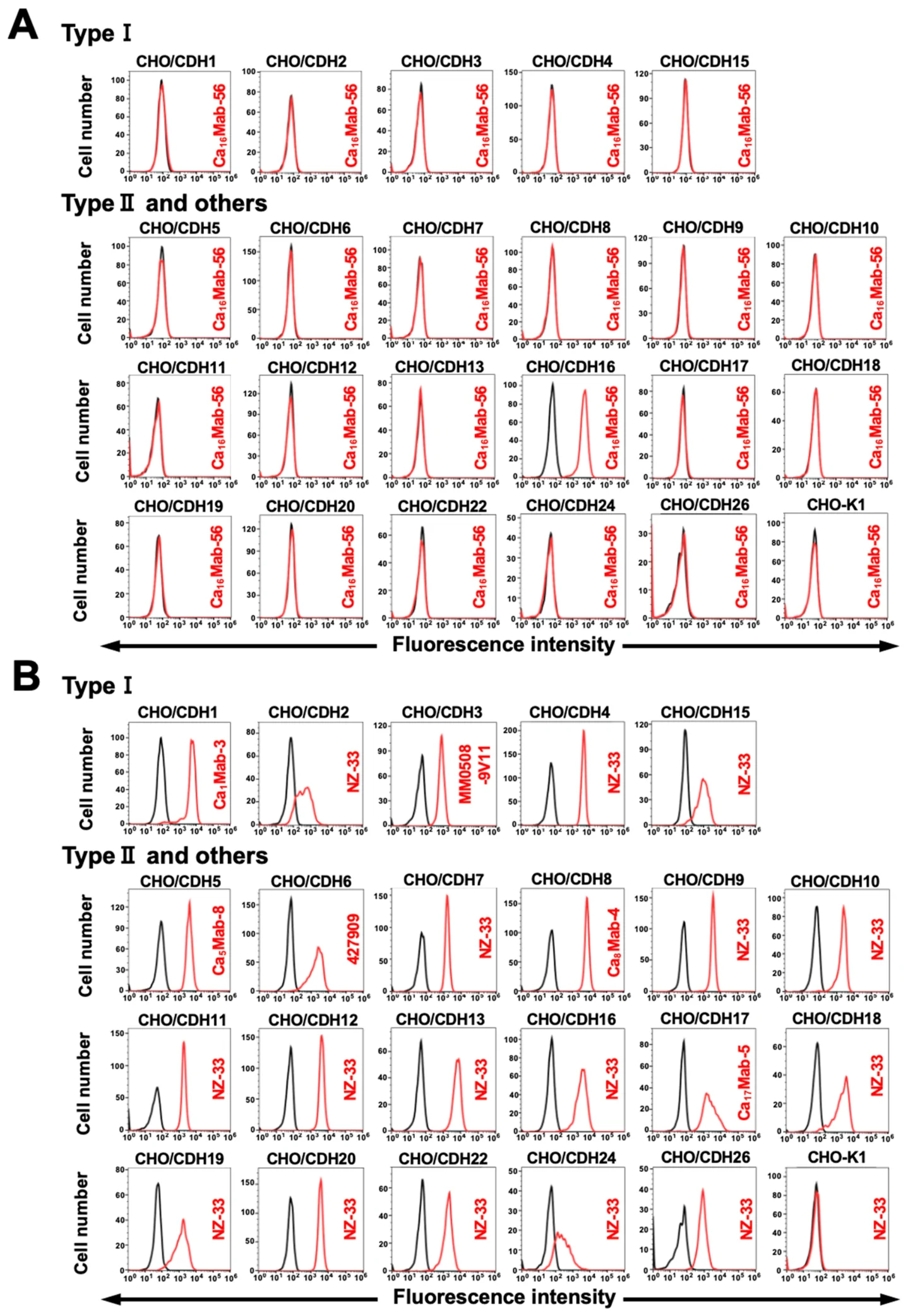
Specificity of Ca_16_Mab-56. (**A**) The type I CDHs, type II CDHs, a truncated CDH, 7D CDHs, and an atypical CDH-overexpressed CHO-K1 were treated with 5 µg/mL of Ca_16_Mab-56 (red) or with control blocking buffer (black, negative control), followed by treatment with anti-mouse IgG conjugated with Alexa Fluor 488. (**B**) Each CDHs expression was confirmed by 1 µg/mL of an anti-CDH1 mAb (clone Ca_1_Mab-3), 1 µg/mL of an anti-CDH3 mAb (clone MM0508-9V11), 1 µg/mL of an anti-CDH6 mAb (clone 427909), 1 µg/mL of an anti-CDH8 mAb (clone Ca_8_Mab-4), 1 µg/mL of an anti-CDH17 mAb (clone Ca_17_Mab-5), and 0.1 µg/mL of an anti-PA16-tag mAb (clone NZ-33) to detect other CDHs, followed by the treatment with anti-mouse IgG conjugated with Alexa Fluor 488. The fluorescence data were collected using the SA3800 Cell Analyzer. The experiments were conducted twice.

Using the CDHs-overexpressed CHO-K1 cell lines [29], the specificity of Ca_16_Mab-56 was investigated. As shown in Figure 3A, Ca_16_Mab-56 recognized CHO/CDH16 but did not cross-react with CHO-K1 overexpressed type I CDHs (CDH1, CDH2, CDH3, CDH4, CDH15), type II CDHs (CDH5, CDH6, CDH7, CDH8, CDH9, CDH10, CDH11, CDH12, CDH18, CDH19, CDH20, CDH22, CDH24), a truncated CDH (CDH13), another 7D CDH (CDH17), and an atypical CDH (CDH26). The cell surface expression of each CDH was confirmed in Figure 3B. Although BT-MCA3873 showed the highest reactivity to CHO/CDH16, the cross-reactivity with CDH7, CDH11, and CDH22 was observed (Supplementary Figure S1). These results indicated that Ca_16_Mab-56 is a CDH16-specific mAb among those CDHs.

### 3.4. Flow Cytometric Analysis of Ca_16_Mab-56 and BT-MCA3873 Against Endogenous CDH16-Expressing Cell Lines

**Figure 4:**
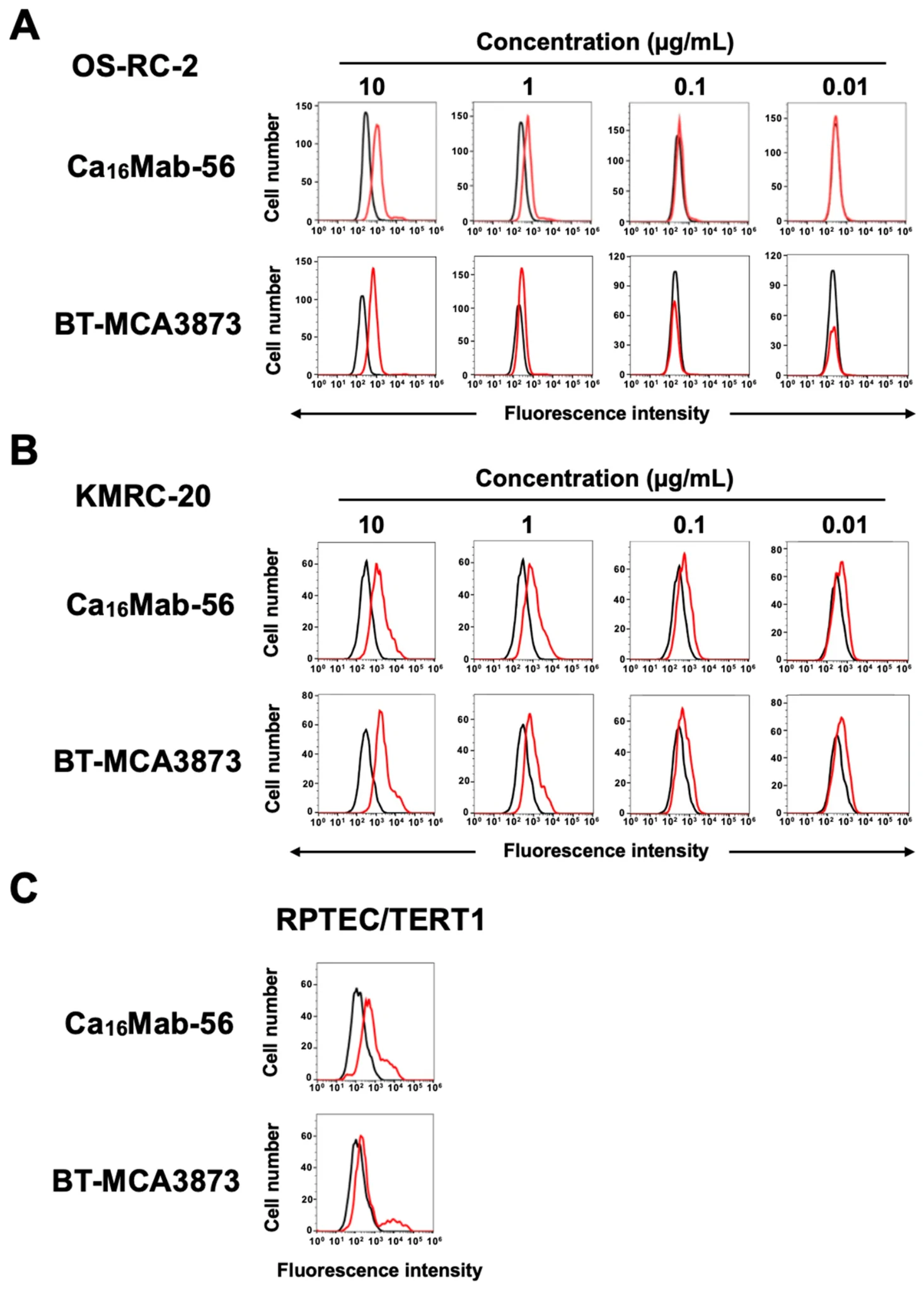
Flow cytometric analysis using Ca_16_Mab-56 and BT-MCA3873 to detect endogenous CDH16. OS-RC-2 (**A**) and KMRC-20 (**B**) were treated with Ca_16_Mab-56 and BT-MCA3873 at the indicated concentrations (red). (**C**) RPTEC/TERT1 were treated with 10 µg/mL of Ca_16_Mab-56 and BT-MCA3873 (red). The black line represents negative control (blocking buffer). These cells were incubated with Alexa Fluor 488-conjugated anti-mouse IgG. Fluorescence data were collected using the SA3800 Cell Analyzer. The experiments were conducted twice.

Expression of endogenous CDH16 in various RCC and normal kidney cell lines was investigated using Ca_16_Mab-56 and BT-MCA3873. Ca_16_Mab-56 and BT-MCA3873 dose-dependently recognized endogenous CDH16 in RCC cell lines OS-RC-2 (Figure 4A) and KMRC-20 (Figure 4B). Additionally, Ca_16_Mab-56 recognized the immortalized human normal kidney epithelial cell line RPTEC/TERT1, but the reactivity was low with BT-MCA3873 (Figure 4C). These results indicated that Ca_16_Mab-56 recognizes endogenous CDH16 in normal kidney epithelial and RCC cell lines.

**Figure 5:**
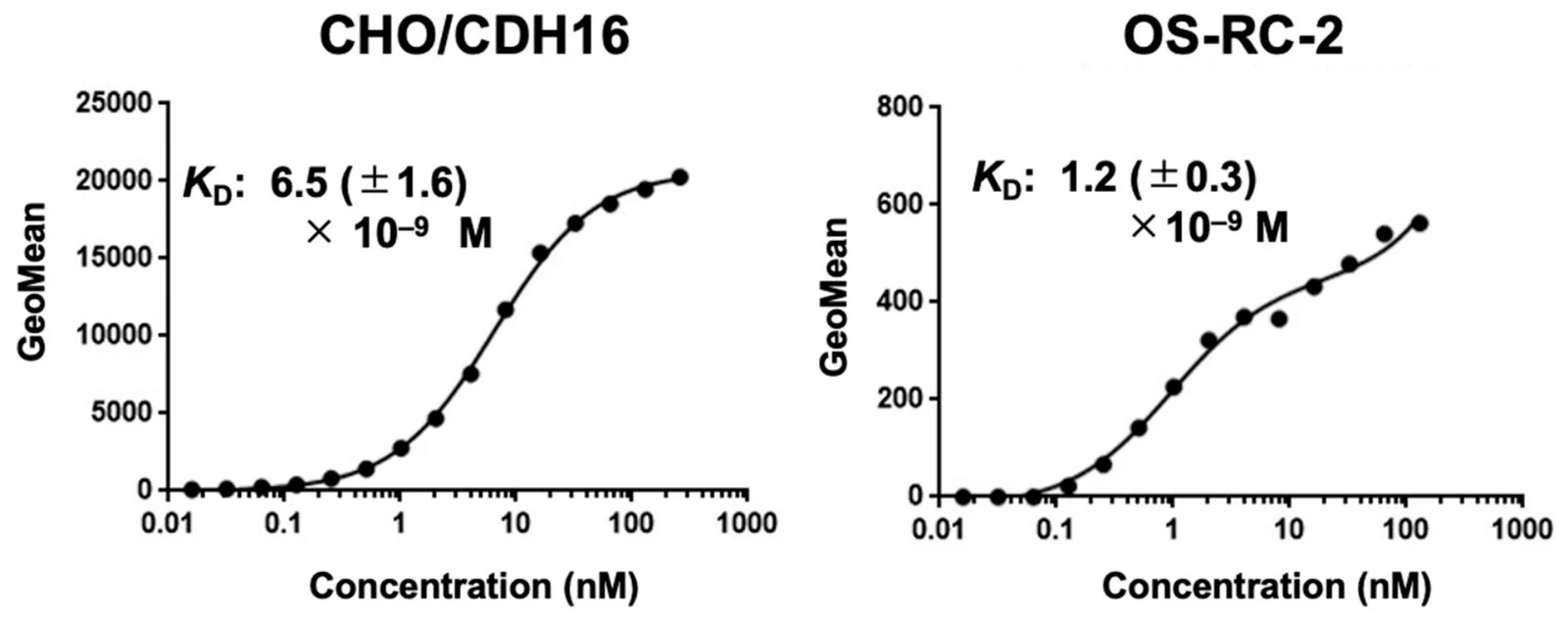
Determination of the binding affinity of Ca_16_Mab-56 by flow cytometry. CHO/CDH16 and OS-RC-2 cells were incubated with serially diluted Ca_16_Mab-56, then reacted with Alexa Fluor 488-conjugated anti-mouse IgG. Geometric mean fluorescence values were measured using the SA3800 Cell Analyzer and FlowJo software. Average *K*_D_ values (±standard deviation) from three independent measurements were calculated using GraphPad PRISM 6. Representative graphs are shown.

The binding affinity of Ca_16_Mab-56 was measured for CHO/CDH16 and OS-RC-2 using flow cytometry. The *K*_D_ values of Ca_16_Mab-56 with CHO/CDH16 and OS-RC-2 were 6.5 (± 1.6) × 10^−9^ M and 1.2 (± 0.3) × 10^−9^ M, respectively (Figure 5). The *K*_D_ value of BT-MCA3873 for CHO/CDH16 could not be determined because the sigmoid curve did not reach a plateau under the same experimental conditions (Supplementary Figure S2). These results showed that Ca_16_Mab-56 has superior binding affinity for exogenous and endogenous CDH16.

### 3.5. Detection of Exogenous and Endogenous CDH16 by Ca_16_Mab-56 and BT-MCA3873 in Western Blotting

**Figure 6:**
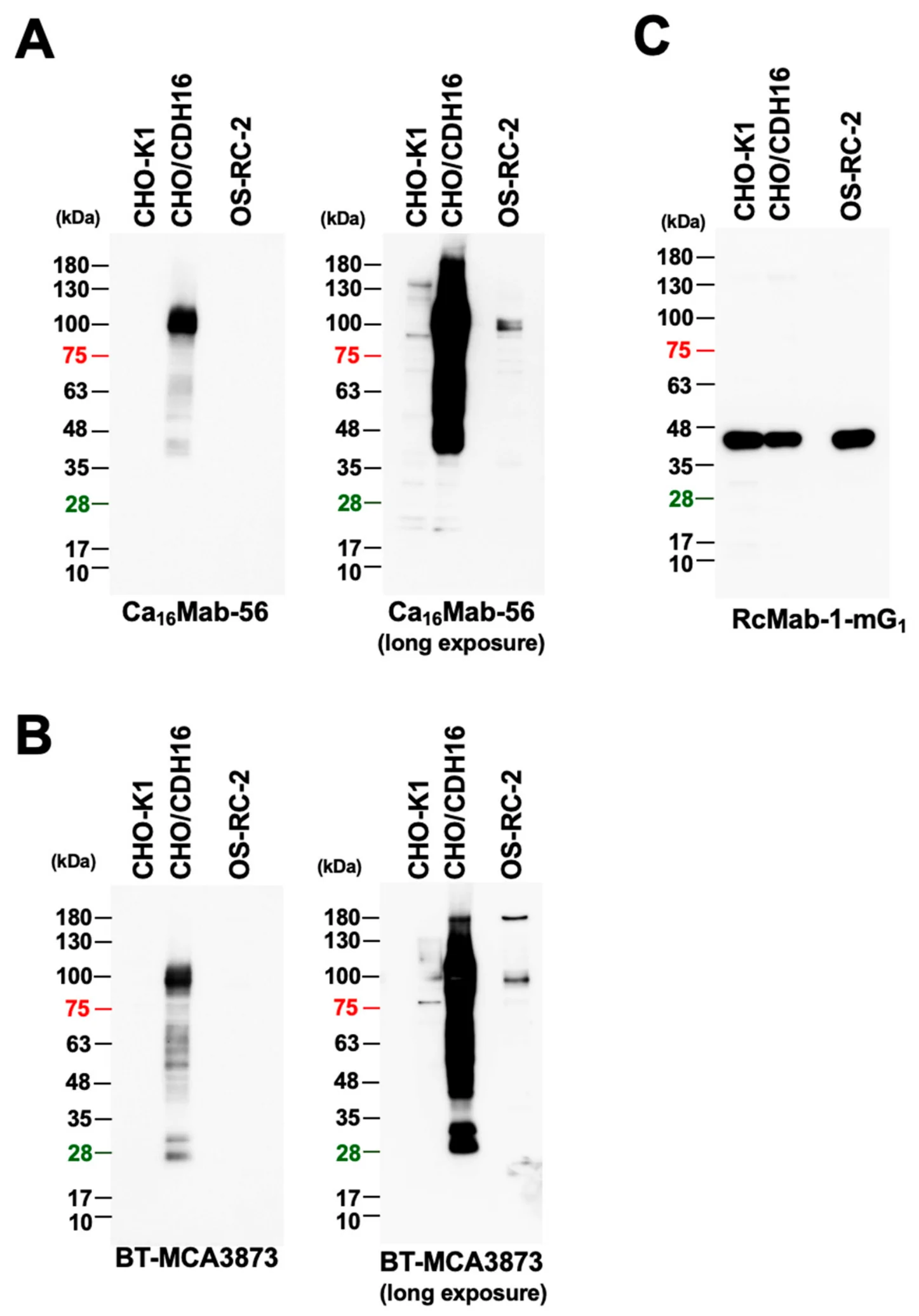
Western blotting using Ca_16_Mab-56 and BT-MCA3873. Cell lysates (10 μg/lane) from CHO-K1, CHO/CDH16, and OS-RC-2 were electrophoresed and transferred to polyvinylidene difluoride membranes. The membranes were incubated with 1 μg/mL of Ca_16_Mab-56 (**A**) or BT-MCA3873 (**B**). (**C**) An anti-IDH1 mAb (clone RcMab-1-mG_1_, 2 μg/mL) was used as an internal control. The membranes were further treated with anti-mouse IgG conjugated with horseradish peroxidase. The experiments were conducted twice.

We next examined whether Ca_16_Mab-56 and BT-MCA3873 can be used in Western blotting. As shown in Figure 6A, Ca_16_Mab-56 detected a band of approximately 100 kDa in CHO/CDH16 cell lysates, whereas no band was detected in parental CHO-K1 cell lysates. Furthermore, Ca_16_Mab-56 detected endogenous CDH16 in OS-RC-2 cell lysates at 100 kDa (Figure 6A). Similar reactivity was observed in CHO/CDH16 cell lysates using BT-MCA3873 (Figure 6B). Moreover, two bands of approximately 100 kDa and 180 kDa were detected in OS-RC-2 cell lysates using BT-MCA3873 (Figure 6B). An anti-IDH1 mAb (clone RcMab-1-mG_1_) was used to show the equal loading (Figure 6C). These results indicated that Ca_16_Mab-56 and BT-MCA3873 can detect exogenous and endogenous CDH16 by Western blotting.

### 3.6. Immunohistochemistry Using Ca_16_Mab-56 in Formalin-Fixed Paraffin-Embedded Cell Blocks

**Figure 7:**
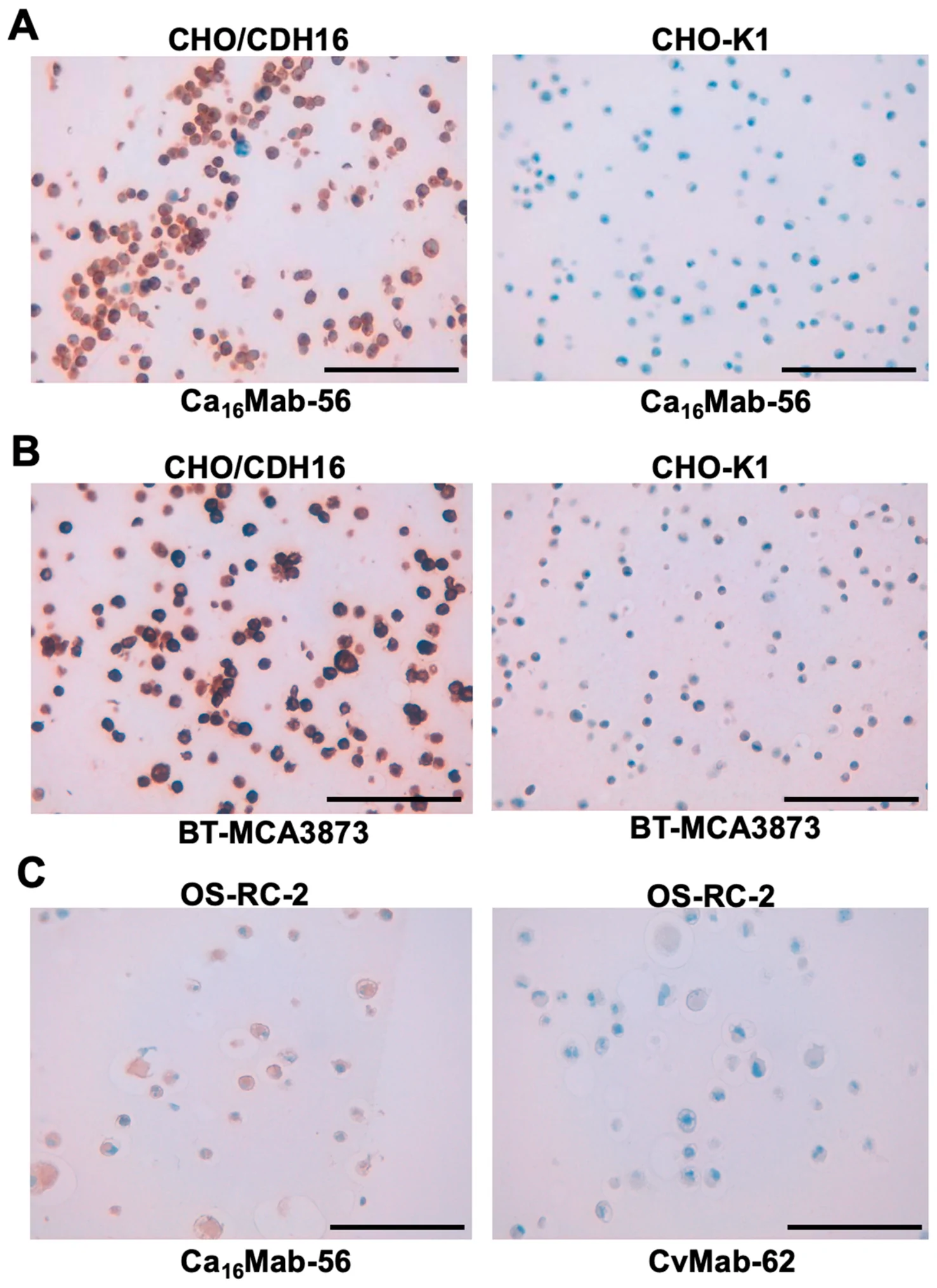
Immunohistochemistry using Ca_16_Mab-56 and BT-MCA3873 in formalin-fixed paraffin-embedded cell blocks. (**A**) CHO/CDH16 and CHO-K1 sections were treated with 1 μg/mL of Ca_16_Mab-56. (**B**) CHO/CDH16 and CHO-K1 sections were treated with 0.2 μg/mL of BT-MCA3873. (**C**) OS-RC-2 sections were treated with 5 μg/mL of Ca_16_Mab-56 or 5 μg/mL of CvMab-62 (IgG_1_ isotype control). Staining was performed using VENTANA BenchMark ULTRA PLUS with the ultraView Universal DAB Detection Kit. Scale bar = 100 μm. Experiments were conducted twice.

We investigated whether Ca_16_Mab-56 and BT-MCA3873 are suitable for IHC in FFPE sections from CHO-K1 and CHO/CDH16. Ca_16_Mab-56 showed clear membranous staining in CHO/CDH16 but not in CHO-K1 (Figure 7A). BT-MCA3873 showed more potent reactivity to CHO/CDH16, but weak reactivity to CHO-K1 was also detected at the lower concentration (Figure 7B). Furthermore, Ca_16_Mab-56 also showed positive staining in OS-RC-2, but an IgG_1_ isotype control mAb (CvMab-62) did not (Figure 7C). These results indicated that Ca_16_Mab-56 can detect both exogenous and endogenous CDH16 of FFPE cell sections in IHC.

### 3.7. Immunohistochemistry Using Ca_16_Mab-56 in Normal Kidney and RCCs

**Figure 8:**
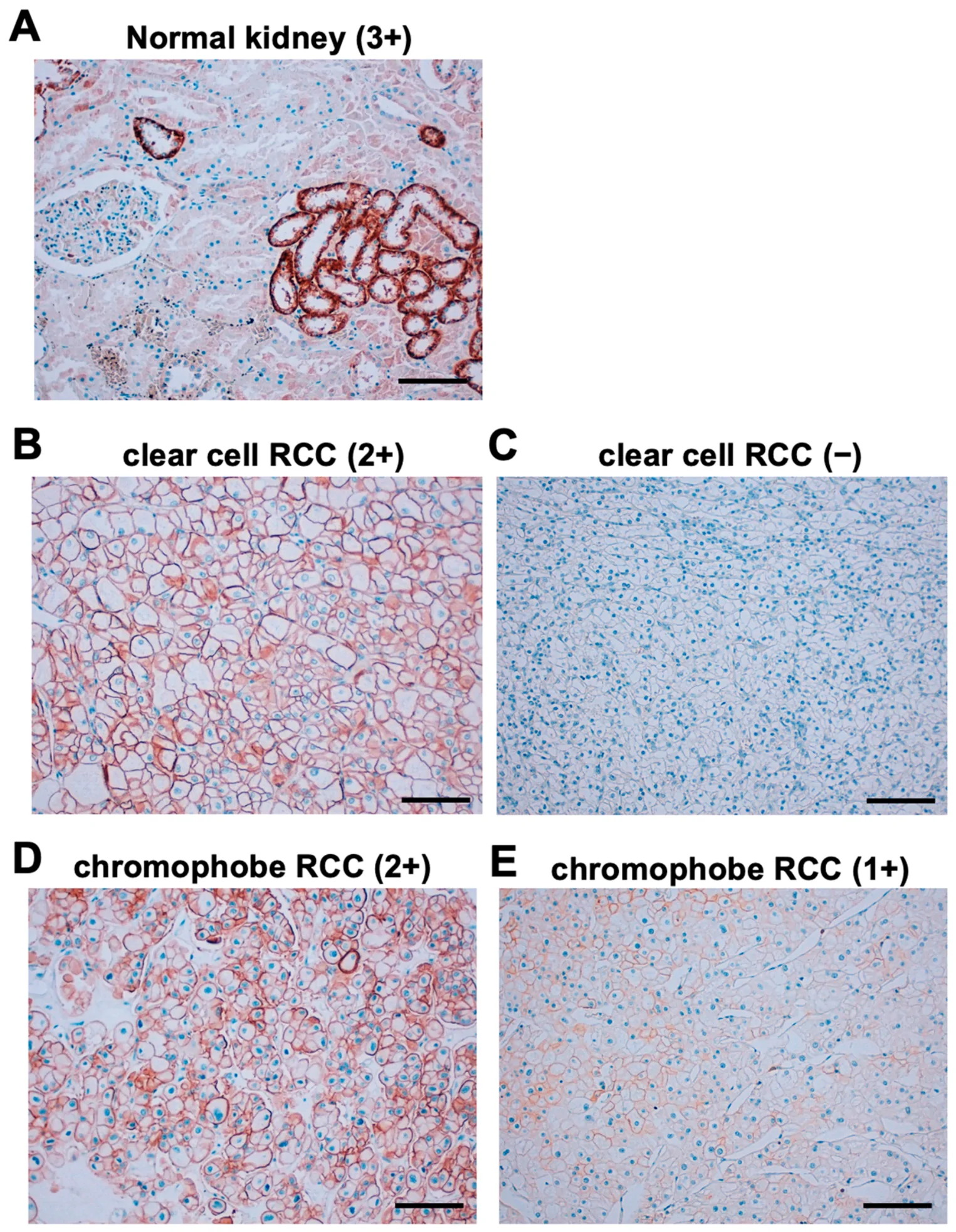
Immunohistochemistry with Ca_16_Mab-56 on a multiple kidney tumor microarray (KD1501a). The microarray was incubated with 5 μg/mL of Ca_16_Mab-56. (**A**) Normal kidney tubular epithelium (strong intensity, 3+). Representative images of clear cell RCC with moderate intensity (2+, (**B**)) and no staining (–, (**C**)) are shown. Representative images of chromophobe RCC with moderate intensity (2+, (**D**)) and weak intensity (1+, (**E**)) are shown. Staining was performed on a VENTANA BenchMark ULTRA PLUS using the ultraView Universal DAB Detection Kit. Scale bar = 100 μm.

CDH16 expression is reported to be restricted to the kidney [18]. We next examined CDH16 expression using Ca_16_Mab-56 in a human normal kidney tissue microarray. Supplementary Figure S3 shows several sections stained with Ca_16_Mab-56. Strong staining was observed in distal tubules, while staining was moderate in proximal tubules with a brush border. Under the same staining conditions, similar strong staining (3+) was observed in a normal kidney from another multiple kidney microarray (KD1501a, Figure 8A). Furthermore, Ca_16_Mab-56 showed clear membranous staining (moderate intensity, 2+) in a case of clear cell RCC (Figure 8B), but most clear cell RCC cases were negative (Figure 8C). In chromophobe RCC, clear membranous staining patterns [moderate intensity, 2+ (Figure 8D) or weak intensity, 1+ (Figure 8E)] were observed. Table 1 summarizes the results of the multiple kidney tumor microarray (KD1501a). Consequently, Ca_16_Mab-56 stained 1 out of 33 cases of clear cell RCC (3%), three out of five cases of chromophobe RCC (60%), and one out of nine cases of papillary renal cell carcinoma (type II) (11%).

## 4. Discussion

In this study, we developed novel anti-CDH16 mAbs (58 clones) by immunizing a mouse with CDH16-overexpressed LN229 cells, followed by high-throughput flow cytometry-based screening (Figure 1). We further evaluated the applications, including flow cytometry, Western blotting, and immunohistochemistry, and found that clone Ca_16_Mab-56 is a versatile mAb for detecting CDH16 across these three applications. Information on the other clones is available on a website (https://www.med-tohoku-antibody.com/topics/antibody_bank.htm, accessed on 23 July 2026). Ca_16_Mab-56 recognized both exogenous and endogenous CDH16 by flow cytometry (Figure 2 and Figure 4). Ca_16_Mab-56 could detect CDH16 in RCC cell lines and in an immortalized human normal kidney epithelial cell line (Figure 4). Importantly, Ca_16_Mab-56 specifically recognized CDH16 without detectable cross-reactivity to 21 other CDHs, including classical type I and type II CDHs, and other types of CDHs (Figure 3). In contrast, the commercially available anti-CDH16 mAb (BT-MCA3873) showed cross-reactivity with CDH7, CDH11, and CDH22 (Supplementary Figure S1), suggesting that careful handling and interpretation of data are essential.

Ca_16_Mab-56 can be used for IHC of cell specimens (Figure 7) and RCC tissues (Figure 8) conducted using an automated slide-staining system, VENTANA BenchMark ULTRA PLUS. This ensures reproducible staining and accurate assessment of CDH16 expression for diagnosis. As with previous reports [18,20,23], CDH16 staining by Ca_16_Mab-56 was predominant in the basolateral membrane and cytosol and was stronger in distal tubules than in proximal tubules in normal kidney (Figure 8A and Supplementary Figure S3). Although the CDH16-positive ratio by Ca_16_Mab-56 in chromophobe RCC was similar, that in clear cell RCC was low compared to the previous observations. Since chromophobe RCC has been suggested to originate from distal tubules [8,19], the CDH16 expression is thought to be reduced during tumor progression. The CDH16 positivity in clear cell RCC was distributed from 0% [8], 14% [19], 17% [22], 30% [30], 82% [20], and 85% [23], which could be explained by the sensitivity, specificity, and epitope of mAbs. An anti-CDH16 mAb (clone 4H6/F9), which recognized the C-terminal cytoplasmic domain of CDH16, was used in the first four studies [8,19,22,30]. In contrast, the detailed immunogens of mAbs were not disclosed in the remaining studies [20,23]. Furthermore, the information about cross-reactivity to other CDHs could not be obtained. Since Ca_16_Mabs were selected by flow cytometry-based screening, most of the Ca_16_Mabs could not be used in IHC. Only a few Ca_16_Mabs, including Ca_16_Mab-56, can be used for IHC, and no cross-reactivity to the other 21 CDHs was confirmed in Ca_16_Mab-56 (Figure 3). Therefore, the positive signal by Ca_16_Mab-56 is considered to represent the recognition of CDH16.

Kidneys are highly enriched in mitochondria and have substantial energy demands due to their high basal metabolic activity. Mitochondrial dysfunction-driven metabolic dysregulation is a defining feature of acute kidney injury [31], chronic kidney disease [32], and kidney aging [33]. In a mouse model, isolation of mitochondria from podocytes or CDH16-positive tubules revealed that mitochondrial function is essential for maintaining podocyte integrity, and that mitochondrial respiration declines more markedly with aging in male podocytes than in female podocytes or kidney tubules, potentially contributing to the development of age-associated glomerulosclerosis [34]. Viable human kidney epithelial cells can be obtained from a kidney apart from RCC after nephrectomy or a kidney biopsy [35,36]. Freshly isolated human proximal tubular epithelial cells (PTECs) from the kidney cortex have been used in tissue engineering and in vitro models of kidney diseases [37,38]. RPTEC/TERT1 cell line, generated by immortalization of PTECs with human telomerase reverse transcriptase (hTERT) [39], weakly expressed CDH16 (Figure 4C). Since CDH16 is abundantly expressed in distal tubular epithelial cells in IHC (Figure 8A and Supplementary Figure S3), distal tubular epithelial cells could be sorted as CDH16-high population using Ca_16_Mab-56. Analysis of CDH16-low PTECs and CDH16-high distal tubular epithelial cells may contribute to the comparison of biological properties, including mitochondrial activity.

In contrast to CDH16, another 7D CDH, CDH17 is expressed almost exclusively in intestinal epithelial cells of the small intestine and colon [40] and is frequently overexpressed in advanced gastric cancer, hepatocellular carcinoma, and colorectal cancers [41,42,43]. In hepatocellular carcinoma, elevated CDH17 expression is associated with activation of Wnt/β-catenin signaling, vascular invasion, and advanced tumor stage [43]. In colorectal cancer, CDH17 promotes tumor progression by activating α2β1 integrin, thereby enhancing cell proliferation and adhesion to type IV collagen [41]. These results highlight the essential roles of CDH17 in promoting tumor progression. Therefore, multiple therapeutic strategies, including mAb monotherapy [44], bispecific Abs [45], antibody–drug conjugates [46], CAR-T [47], and CAR-NK cell [48] therapies have been developed for the treatment of CDH17-positive tumors and evaluated in clinical trials [49]. However, anti-CDH16 therapy has not been developed or evaluated. We have successfully cloned Ca_16_Mab-56 cDNA, and an isotype-converted human IgG_1_-type Ca_16_Mab-56 will be generated. We will investigate antitumor efficacy using an in vitro antibody-dependent cellular cytotoxicity assay and tumor xenograft models.

As shown in Figure 8A, Supplementary Figure S3, and previous studies, CDH16 is predominantly localized to the kidney distal tubules, which predicts anti-CDH16 therapy-associated toxicities and adverse effects in the kidney. To improve the therapeutic window, we previously developed cancer-specific monoclonal antibodies (CasMabs), including the anti-HER2 CasMab H_2_CasMab-2 [28], which selectively recognized cancer cells and showed potent antitumor activity in preclinical models [50], leading to a phase I clinical trial (NCT06241456). These findings highlight the importance of developing CDH16-specific CasMabs. As shown in Figure 4, Ca_16_Mab-56 reacted with RCCs (OS-RC-2 and KMRC-20) and with normal kidney epithelial cells (RPTEC/TERT1). We will screen other Ca_16_Mabs and select anti-CDH16 CasMabs which react with the RCCs but not RPTEC/TERT1. The strategy is thought to be important to reduce adverse effects and improve the therapeutic window.

## Figures and Tables

**Table 1:** Immunohistochemistry of a multiple kidney tumor microarray (KD1501a) by Ca_16_Mab-56.

No.	Age	Sex	Pathology Diagnosis	TNM	Ca_16_Mab-56
1	67	M	Clear cell carcinoma	T3N0M0	−
2	65	M	Clear cell carcinoma	T3N0M0	−
3	60	M	Clear cell carcinoma	T2N0M0	−
4	57	F	Clear cell carcinoma	T2N0M0	−
5	40	F	Clear cell carcinoma	T2N0M0	−
6	40	F	Clear cell carcinoma	T2N0M0	−
7	84	M	Clear cell carcinoma	T2N0M0	−
8	51	F	Clear cell carcinoma	T2N0M0	2+
9	62	F	Clear cell carcinoma	T2N0M0	−
10	60	F	Clear cell carcinoma	T2N0M0	−
11	67	M	Clear cell carcinoma	T2N0M0	−
12	45	M	Clear cell carcinoma	T2N0M0	−
13	65	M	Clear cell carcinoma	T2N0M0	−
14	54	M	Clear cell carcinoma	T2N0M0	−
15	51	M	Clear cell carcinoma	T2aN0M0	−
16	54	M	Clear cell carcinoma	T2N0M0	−
17	76	M	Clear cell carcinoma	T2N0M0	−
18	58	M	Clear cell carcinoma	T2N0M0	−
19	50	F	Clear cell carcinoma	T2N0M0	−
20	36	M	Clear cell carcinoma	T2N0M0	−
21	70	M	Clear cell carcinoma	T2N1M0	−
22	-	-	Clear cell carcinoma	T2aN0M0	−
23	63	M	Clear cell carcinoma	T2N0M0	−
24	70	M	Clear cell carcinoma	T2N0M0	−
25	54	M	Clear cell carcinoma	T2aN0M0	−
26	62	M	Clear cell carcinoma	T2N0M0	−
27	55	M	Clear cell carcinoma	T2N0M0	−
28	33	F	Clear cell carcinoma	T2N0M0	−
29	58	M	Clear cell carcinoma	T2aN0M0	−
30	71	F	Clear cell carcinoma	T2N0M0	−
31	49	M	Clear cell carcinoma	T2N0M0	−
32	54	M	Clear cell carcinoma	T3N0M0	−
33	52	M	Invasive urothelial carcinoma of renal pelvis	T2N0M0	−
34	77	M	Invasive urothelial carcinoma of renal pelvis	T3N0M0	−
35	23	M	Invasive urothelial carcinoma of renal pelvis	T2N0M0	−
36	56	M	Invasive urothelial carcinoma of renal pelvis	T2N1M0	−
37	73	M	Invasive urothelial carcinoma of renal pelvis	T1N0M0	−
38	58	M	Invasive urothelial carcinoma of renal pelvis	T3N0M0	−
39	76	M	Invasive urothelial carcinoma of renal pelvis	T2N1M0	−
40	51	M	Invasive urothelial carcinoma of renal pelvis	T2N0M0	−
41	−	−	Invasive urothelial carcinoma of renal pelvis	T3N0M0	−
42	42	M	Invasive urothelial carcinoma of renal pelvis	T3N0M0	−
43	68	F	Chromophobe cell carcinoma	T1aN0M0	1+
44	52	M	Clear cell carcinoma	T2N0M0	−
45	61	M	Chromophobe cell carcinoma	T1N0M0	2+
46	43	M	Chromophobe cell carcinoma	T2N0M0	−
47	55	F	Chromophobe cell carcinoma	T2N0M0	−
48	39	M	Chromophobe cell carcinoma	T1aN0M0	1+
49	54	M	Papillary renal cell carcinoma (type II)	T2aN0M0	1+
50	57	F	Papillary renal cell carcinoma (type II)	T2N0M0	−
51	41	M	Papillary renal cell carcinoma (type II)	T1N0M0	−
52	48	M	Papillary renal cell carcinoma (type II)	T1bN0M0	−
53	53	M	Papillary renal cell carcinoma (type II)	T1N0M0	−
54	34	F	Papillary renal cell carcinoma (type II)	T2aN1M0	−
55	68	M	Papillary renal cell carcinoma (type II)	T1bN0M0	−
56	53	M	Papillary renal cell carcinoma (type II)	T1bN0M0	−
57	63	M	Papillary renal cell carcinoma (type II)	T1bN0M0	−
58	52	M	Squamous cell carcinoma (nephritis)	T1N0M0	−
59	58	F	Squamous cell carcinoma	T3N1M0	−
60	45	M	Squamous cell carcinoma	T2N0M0	−
61	56	M	Squamous cell carcinoma	T1N0M0	−
62	56	M	Mixed renal cell carcinoma	T3N0M0	−
63	57	M	Mixed renal cell carcinoma	T2aN0M0	−
64	45	M	Renal collecting duct carcinoma	T1N0M0	−
65	43	F	Diffuse T cell lymphoma	−	−
66	56	M	Diffuse T cell lymphoma	−	−
67	58	M	Sarcomatoid carcinoma	T2N1M0	−
68	64	M	Sarcomatoid carcinoma	T2N0M0	−
69	51	F	Leiomyosarcoma	−	−
70	48	M	Angiomyolipoma	−	−
71	−	−	Angioleiomyoma	−	−
72	57	M	Adjacent normal kidney tissue	−	−
73	43	M	Kidney tissue	−	3+
74	37	M	Kidney tissue	−	2+
75	44	M	Kidney tissue	−	3+

−, No stain; 1+, Weak intensity; 2+, Moderate intensity; 3+, Strong intensity.

## Data Availability

The data presented in this study are available in the article.

## Supplementary Materials

The following supporting information can be downloaded at: https://www.mdpi.com/article/10.3390/antib15050083/s1, Figure S1: Specificity of BT-MCA3873. Figure S2: Determination of binding affinity of BT-MCA3873 by flow cytometry. Figure S3: Immunohistochemistry using Ca_16_Mab-56 (5 µg/mL) in a human normal kidney tissue microarray.
