# Supplementary material for: Ca16Mab-56, a Novel Anti-Cadherin-16/Ksp-Cadherin Monoclonal Antibody for Multiple Applications
Source: Antibodies (Basel). 2026 Sep 7;15(5):83. doi: 10.3390/antib15050083 (PMC13600073; doi:10.3390/antib15050083)
Supplement: Supplementary file 1 [file antibodies-15-00083-s001.zip › antibodies-4496011-supplementary.pdf]

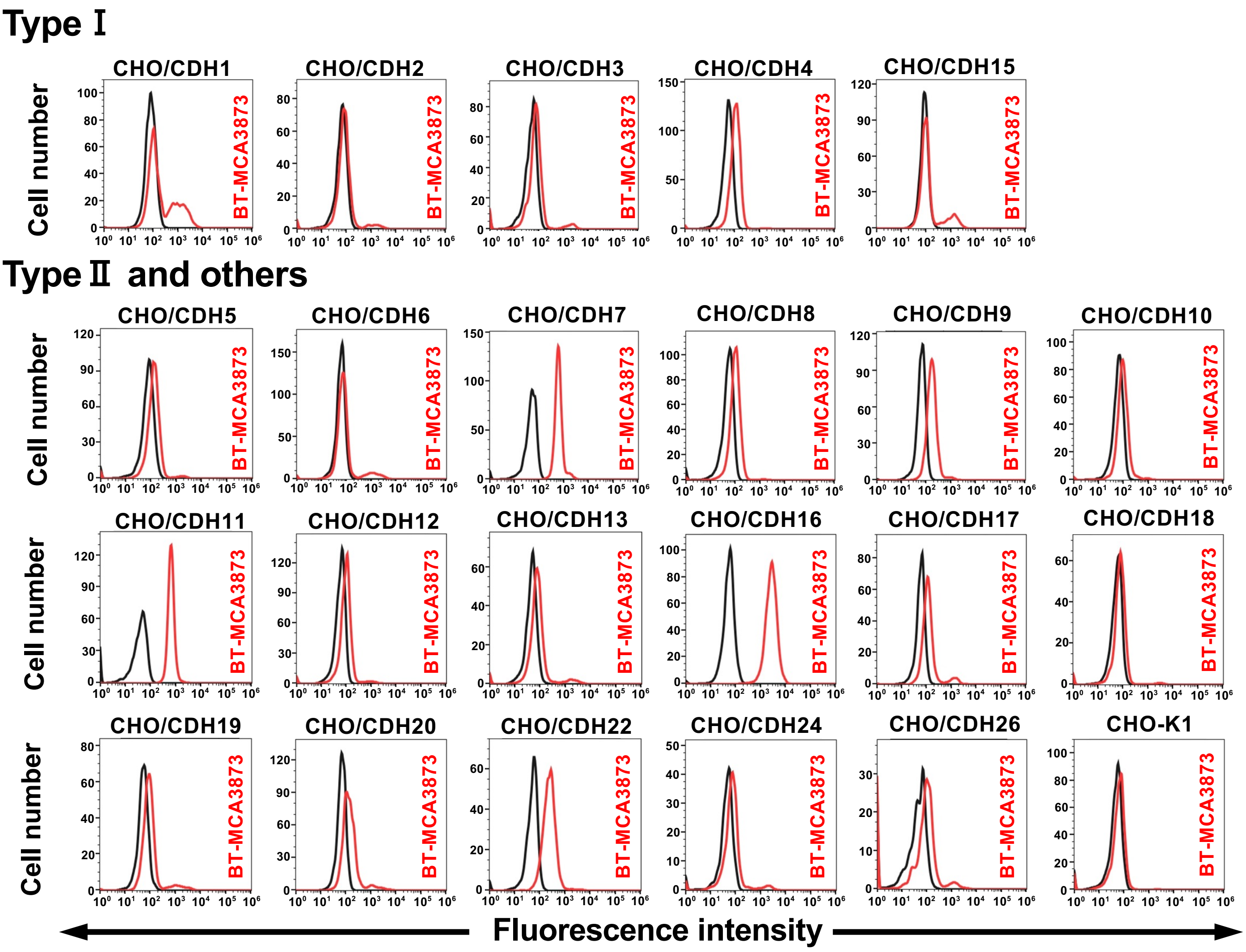

Supplementary Figure S1. Specificity of BT-MCA3873. The type I CDHs, type II CDHs, a truncated CDH, 7D CDHs, and an atypical CDH-overexpressed CHO-K1 were treated with 5 µg/mL of BT-MCA3873 (red) or with control blocking buffer (black, negative control), followed by treatment with anti-mouse IgG conjugated with Alexa Fluor 488. The fluorescence data were collected using the SA3800 Cell Analyzer. Each cadherin expression was confirmed in Figure 3B.

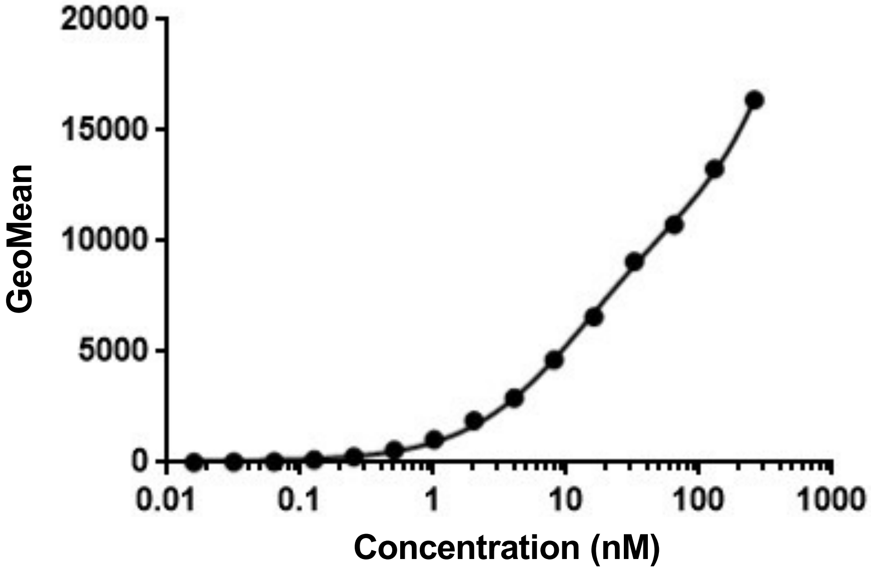

Supplementary Figure S2. Determination of binding affinity of BT-MCA3873 by flow cytometry. CHO/CDH16 were suspended in serially diluted Ca_{16}Mab-56. Then, cells were reacted with Alexa Fluor 488-conjugated anti-mouse IgG. The geometric mean fluorescence values were obtained using the SA3800 Cell Analyzer and FlowJo software. The K_D value of BT-MCA3873 for CHO/CDH16 could not be determined because the sigmoid curve did not reach a plateau

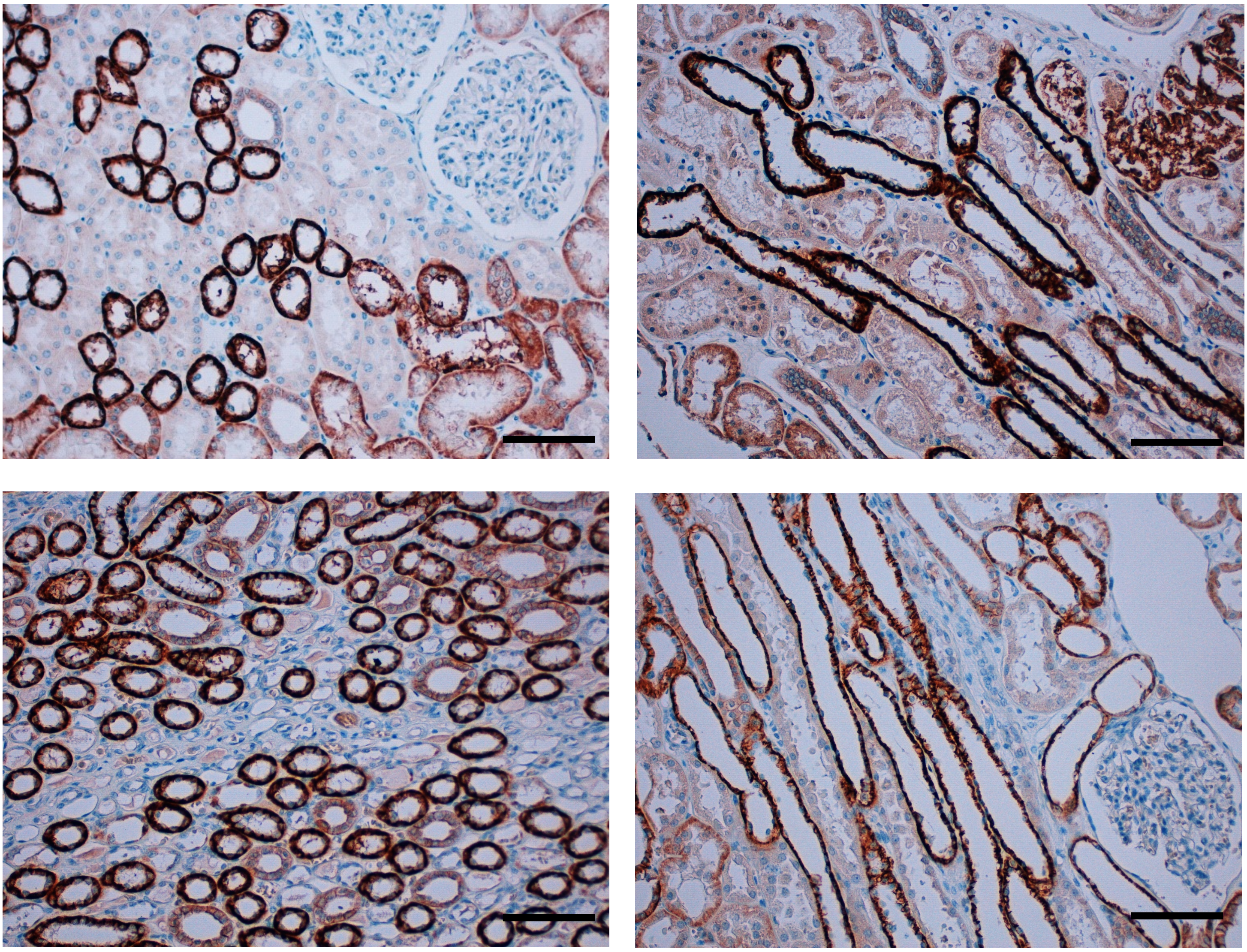

Supplementary Figure S3. Immunohistochemistry using Ca_{16}Mab-56 (5 μg/mL) in a human normal kidney tissue microarray. The staining was performed using VENTANA BenchMark ULTRA PLUS with the ultraView Universal DAB Detection Kit. Scale bar = 100 μm.
